# Identifying structural domains of proteins using clustering

**DOI:** 10.1186/1471-2105-13-286

**Published:** 2012-11-01

**Authors:** Howard J Feldman

**Affiliations:** 1 Chemical Computing Group, Inc., 1010 Sherbrooke St. W., Suite 910, Montreal, Quebec, H3A 2R7, Canada

**Keywords:** Domain assignment, Agglomerative clustering, Average-linkage, Structural domain

## Abstract

**Background:**

Protein structures are comprised of modular elements known as domains. These units are used and re-used over and over in nature, and usually serve some particular function in the structure. Thus it is useful to be able to break up a protein of interest into its component domains, prior to similarity searching for example. Numerous computational methods exist for doing so, but most operate only on a single protein chain and many are limited to making a series of cuts to the sequence, while domains can and do span multiple chains.

**Results:**

This study presents a novel clustering-based approach to domain identification, which works equally well on individual chains or entire complexes. The method is simple and fast, taking only a few milliseconds to run, and works by clustering either vectors representing secondary structure elements, or buried alpha-carbon positions, using average-linkage clustering. Each resulting cluster corresponds to a domain of the structure. The method is competitive with others, achieving 70% agreement with SCOP on a large non-redundant data set, and 80% on a set more heavily weighted in multi-domain proteins on which both SCOP and CATH agree.

**Conclusions:**

It is encouraging that a basic method such as this performs nearly as well or better than some far more complex approaches. This suggests that protein domains are indeed for the most part simply compact regions of structure with a higher density of buried contacts within themselves than between each other. By representing the structure as a set of points or vectors in space, it allows us to break free of any artificial limitations that other approaches may depend upon.

## Background

It is well understood that proteins are made up of structural and functional subunits or 'domains'. Ever since domains were first described [1], numerous methods have been proposed to identify domains within protein structures. These approaches can vary widely depending on whether the assignments are made from sequence alone or from the 3D structure, and often involve partial or complete manual intervention. The domain identification problem is somewhat unique in structural biology in that it is at least in some cases subjective. Different authors have different, though not mutually exclusive, ideas about what a domain should be - a functional unit which is reused over and over [2]; a segment of a structure which has been conserved and reused genetically across different families of proteins [3]; or simply a compact region of the protein where intra-atom contacts outweigh contacts to atoms outside the domain, for rapid self-assembly [1]. Domain definitions are also separated into 'genetic domains' which may be comprised of pieces from multiple chains, and regular ones which are completely contained within a single chain.

As a result of these different paradigms, there still does not exist a precise definition for a protein domain, nor do experts always agree on the number or location of domains within a given structure. This makes it extremely difficult to come up with a fully automated algorithm, then, to assign domain boundaries. That said, the SCOP [4] and CATH [5] databases are typically used for the problem. We found that these agree only 80% of the time on number of domains however, over 75,500 chains that they have in common (SCOP 1.75 and CATH 3.4.0, data not shown)! Despite these problems, splitting a protein into domains is often desirable. For example when performing homology modelling, one often seeks a template to model parts of the structure from. In this case it makes the most sense to find and use similar domains from known structures, which may provide useful templates when searching for similarity to the entire chain may not. Knowledge of domain boundaries can also be used to improve the accuracy of sequence alignments. Many different approaches have been used to split proteins into domains, and these can be divided into sequence-based and structure-based approaches.

Sequence-based domain identification usually involves comparing the sequence in question to a database of protein sequences where the domains have already been defined (such as SCOP) using an alignment tool such as BLAST [6]. More advanced methods such as HMMER [7] make use of multiple sequence alignments of domain families, such as those compiled by InterPro [3], and use Hidden Markov Models (HMM) or other approaches to compare a query sequence against them, recording hits to the various domain families. Examples of sequence-based domain databases include PFAM [8] and SMART [9]. These methods work quite well when sequence identity to known folds is medium to high (above 35% or so) but they fail on novel or unusual folds, or those with only very distant homologs. The precise boundaries may be off by quite a bit as well if there are large insertions or deletions in the sequence relative to the rest of the family.

Structure-based algorithms should in theory be simple and straightforward, and often to the human eye it is obvious where domain boundaries should be drawn when viewing a 3D structure. Nevertheless, it has proved a difficult computational problem and no automated algorithm agrees more than about 80% of the time with SCOP or CATH assignments. A wide variety of methods exist, some based on graph theory and contact maps, some based on secondary structure layout. Some allow only single cuts to be made resulting in domains made of contiguous segments only and a maximum of 3 or 4 domains per chain, others do not have this restriction. PUU [10] builds a contact matrix and tries to maximize interactions within each unit and minimize them between units, through a series of cuts to the sequence. PDP [11] also attempts to make a series of cuts to maximize interactions but normalized the contact count by the expected number of contacts, based on surface area of the proposed domain. DDOMAIN [12] is also based on a series of recursive cuts to try to maximize intra-domain contacts, and also employs a pairwise statistical potential instead of a simple contact count which slightly improves performance. DomainParser [13,14] uses network flow algorithms, hydrophobic moment profile and neural networks to produce its domain partitioning. NCBI's VAST algorithm [15,16], though not fully described anywhere, makes use of domains identified as compact structural units within protein 3D structures using purely geometric criteria. DomainICA [17] uses graph theory with secondary structure elements as the nodes and edges determined by proximity. The algorithm partitions the graph to maximize cycle distributions, and its simplicity is appealing. dConsensus [18] provides a means for rapidly comparing assignments by the different approaches.

Despite the number of algorithms that have been described, most of comparable performance, it seems each has certain disadvantages. As mentioned some methods cannot deal with domains comprised of multiple contiguous segments, and most cannot deal with genetic domains (those with pieces from multiple chains). Some methods are very slow, and some cannot place boundaries midway through secondary structure elements. This study investigates a novel, intuitive algorithm for domain identification by simply clustering α-carbon positions or secondary structure vectors in space. It is very fast, taking under one second for all but the largest proteins, and intuitively obvious. By its nature it has no maximum number of domains it can define, nor limitation on where domain boundaries can occur. Even domains comprised of pieces from multiple chains, such as when domain swapping occurs [19,20], are detected without changes to the algorithm.

## Results

Two distinct but related algorithms were studied, as described in Methods: the α-carbon based algorithm (CA) and the secondary structure element based algorithm (SS). Both make use of average-linkage clustering to produce and then cut a dendrogram; they differ only in the objects that they cluster. The main data set used to optimize the algorithms was the ASTRAL30 set, consisting of 8792 domains in 7178 non-redundant protein chains. Only 7076 of these chains actually still existed in the current Protein Data Bank (PDB) however and so comprised the training set used in this study for the CA algorithm. Only 6841 of these had sufficient secondary structure to use the SS algorithm, so this slightly smaller training set was used in that case. Both algorithms have only two adjustable parameters: the minimum value for cutting the cluster dendrogram, m, and the step size to determine whether to make a cut, s.

For the CA algorithm, a range of these values were tested, and the performance on the training set recorded for each of 1-, 2-, 3-, and 4-domain chains, summarized in Table 1. An assignment was considered correct when it agreed with SCOP, since ASTRAL is based on the SCOP domain database. The Matthews Correlation Coefficient (MCC) was also computed, which gives a statistically less biased measure (compared to percentage correct) of classification success given the large proportion of single domain proteins in the data set. The most obvious effect was that increasing m or s increased the success rate on single-domain proteins, but generally decreased success in the multiple-domain assignments. This is logical as larger values of m and s make it less likely that a cut will be made in the dendrogram, so that a single domain is assigned more often. Assigning a single domain all of the time would of course result in 100% success in single domain protein assignments and 75% success overall which corresponds to the fraction of single domain proteins in the set – as good as any of the parameter sets tested here – however the MCC would be poor. Thus it is important not to put too much value on the overall assignment success, as tempting as it may be to do so. A random assigner was also employed, which chose a number of domains based on the distribution of domains-per-chain in SCOP 1.75, and simply split the sequence equally along its length. As seen in Table 1, this random approach did quite well on single domain proteins but quite poorly on the multi-domain proteins – the CA algorithm clearly does better than chance. The values of m=22Å, s=5Å were chosen because they gave the best compromise of success rates and MCC for multi-domain proteins while still having reasonable performance on the single-domain ones. Only 4% of the 2-domain proteins, and no structures with more than 2 domains, in the ASTRAL set had their corresponding cut in the dendrogram at a value of m < 22Å so this is a reasonable choice.

**Table 1:** CA algorithm assignment success

**m**	**s**	**1-domain**	**2-domain**	**3-domain**	**4-domain**	**Overall**^**1**^
15	5	70% (0.49)	59% (0.34)	47% (0.28)	35% (0.21)	67%
22	5	75% (0.52)	58% (0.35)	45% (0.29)	32% (0.21)	69%
30	5	88% (0.52)	46% (0.37)	32% (0.31)	22% (0.21)	76%
22	3	63% (0.50)	49% (0.24)	42% (0.18)	32% (0.13)	58%
22	5	75% (0.52)	58% (0.35)	45% (0.29)	32% (0.21)	69%
22	8	87% (0.44)	44% (0.33)	26% (0.27)	14% (0.16)	75%
Random	(see text)	76% (≈0)	15% (≈0)	2% (≈0)	0% (≈0)	60%

Only buried α-carbons were clustered, and adjacency constraint was enforced in all cases. Success given on the ASTRAL30 data set as a function of m and s. Matthews correlation coefficient is given in parentheses. ^1^Total, regardless of number of domains.

Similar runs for the SS algorithm are shown in Table 2. Again increasing m and s generally improved single domain success at the cost of multi-domain assignments. For this method complete-linkage clustering was also tested (in addition to average-linkage), and two distance metrics to be used for clustering were tested: closest approach distance of the secondary structure elements, and midpoint distance. While results were comparable, the average-linkage using midpoint distance performed best on multi-domain proteins, with m=22Å and s=5Å having the best compromise on single- and multi-domain success rates. These settings were used for the remainder of the study.

**Table 2:** SS algorithm assignment success

**Linkage**	**metric**	**m**	**s**	**1-domain**	**2-domain**	**3-domain**	**4-domain**
Average	midpt	19	5	71% (0.52)	60% (0.35)	47% (0.28)	34% (0.19)
**Average**	**midpt**	**22**	**5**	**75% (0.55)**	**60% (0.38)**	**46% (0.31)**	**34% (0.21)**
Average	midpt	25	5	80% (0.56)	58% (0.40)	43% (0.32)	28% (0.20)
Average	midpt	22	3	68% (0.53)	51% (0.29)	41% (0.20)	35% (0.15)
**Average**	**midpt**	**22**	**5**	**75% (0.55)**	**60% (0.38)**	**46% (0.31)**	**34% (0.21)**
Average	midpt	22	7	84% (0.53)	55% (0.40)	38% (0.33)	18% (0.18)
**Average**	**closest**	**22**	**3**	**79% (0.55)**	**51% (0.51)**	**40% (0.41)**	**27% (0.27)**
Average	closest	22	4	81% (0.54)	52% (0.38)	39% (0.28)	28% (0.19)
Complete	midpt	40	5	71% (0.51)	45% (0.25)	38% (0.20)	22% (0.10)
**Complete**	**midpt**	**40**	**7**	**73% (0.51)**	**48% (0.28)**	**38% (0.23)**	**23% (0.13)**
Complete	midpt	40	9	77% (0.50)	50% (0.32)	38% (0.26)	18% (0.12)
Complete	midpt	36	7	67% (0.49)	47% (0.24)	37% (0.19)	24% (0.11)
**Complete**	**midpt**	**38**	**7**	**70% (0.50)**	**47% (0.26)**	**38% (0.21)**	**23% (0.11)**
Complete	midpt	42	7	76% (0.51)	49% (0.31)	38% (0.25)	23% (0.14)

Given on the ASTRAL30 data set as a function of m and s. Linkage refers to the clustering technique used in determining the domains. Metric is either *midpt*, meaning distances between secondary structure elements were taken between their midpoints, or *closest*, meaning the closest approach distance was used. The optimal combination of m and s are shown in bold for each section of the table. Matthews correlation coefficient is given in parentheses.

For each algorithm we also tested removing the adjacency constraint - i.e. enforcing a distance of 4Å for Cαs in the same secondary structure element, for the CA algorithm, or a distance of 4Å between consecutive secondary structure elements in the SS algorithm. In both cases removing this had a slight detrimental effect on the success rate (1-2% overall, not shown), so the constraints were left in.

For the CA algorithm, initial tests were done clustering all α-carbons. Using the buried α-carbons only (see Methods) resulted in marked improvement however, increasing single, 2-, 3- and 4-domain proteins from 70%, 55%, 41% and 24% (65% overall) to 75%, 58%, 45% and 32%, respectively (69% overall success). Focusing only on the more buried residues helps make the domain boundaries more clear to the clustering algorithm and so became a permanent part of the algorithm.

The effect of the ‘gold standard’ chosen was also investigated. As mentioned, the success rates in Tables 1 and 2 all used SCOP as the source for the correct answer to each domain splitting problem. However, switching to CATH instead (and discarding the few that were not in CATH) increased the success for the SS algorithm at single domain proteins by 8%, and 2-domain proteins by 7%, while 3- and 4-domain success rates remained about the same (Table 3). Overall success increases from 70% to 74%. Thus the SS algorithm agrees better with CATH than SCOP. This is to be expected since SCOP tends to assign domains with some regard to function, while CATH, like the algorithms in this study, looks at domains from a more structural perspective. If we allow the assignments to match either SCOP or CATH, when they differ, performance increases even further by 0%, 8%, 17% and 21% on 1-, 2-, 3-, and 4-domain proteins respectively (5% overall improvement). Lastly as an interesting test, if we choose m and s to produce the same number of clusters as that given by SCOP and compare to SCOP, so that we are only judging the boundary assignments of the algorithm (the only failures were when the overlap was less than 75%), we see 99%, 86%, 71% and 75% success on 1-, 2-, 3- and 4-domain proteins respectively (95% overall). This is the best we can hope to achieve with perfect choice of cut for every structure. Any further improvement in the algorithm would need to come from better choice of clustering technique. This indicates that the method chooses well where to cut, once the number of cuts to make is known.

**Table 3:** Performance of assignment algorithms as a function of choice of correct answer

**Algorithm**^**1**^	**Correct answer**	**1-domain**	**2-domain**	**3-domain**	**4-domain**	**Overall**^**2**^
SS	SCOP	75%	60%	46%	34%	70%
SS	CATH	83%	67%	47%	34%	74%
SS	SCOP or CATH^3^	83%	75%	64%	55%	79%
SS	SCOP (given)^4^	99%	86%	71%	75%	95%
CA	SCOP	75%	58%	45%	32%	69%
CA	CATH	81%	67%	45%	41%	73%
CA	SCOP or CATH^3^	82%	74%	64%	60%	79%
CA	SCOP (given)^4^	100%	84%	81%	69%	95%

All runs are with m=22Å and s=5Å, and with adjacency constraint enforced, on the ASTRAL30 data set. ^1^CA refers to the α-carbon based algorithm and SS the secondary structure based one. ^2^Total, regardless of number of domains. ^3^Where SCOP and CATH differ, the choice which matched closest to our assignment was chosen in these runs. ^4^The algorithms were forced to cut into the number of domains specified by SCOP for each structure.

Doing the same for the CA algorithm (Table 3), again it was found that when comparing to CATH instead of SCOP, success on single domain proteins increased by 6% and 2-domain and 4-domain proteins each had 9% higher success, while 3-domain proteins were largely unchanged (overall improved by 4% to 73%). So as before, the CA algorithm produces assignments which are more in line with the philosophy adopted by CATH. Allowing the assignments to match either SCOP or CATH when they differ yields significant further increases of 1%, 7%, 19% and 19% for 1-, 2-, 3- and 4-domain proteins respectively (6% overall improvement) and given the number of domains to test the quality of boundary assignments resulted in 100%, 84%, 81% and 69% for 1-, 2-, 3- and 4-domain proteins respectively, or 95% overall. These results were very comparable to those found with the SS algorithm.

Table 4 compares the above results with some of the best available domain assignment algorithms currently available, as well as a random assigner, on the ASTRAL30 database. DDomain offers three assignments using different sets of parameters, but the AUTHORS parameters performed best so only these are reported. All the algorithms clearly perform better than random, and all have very similar performance within a few percentage points of each other, making it difficult to single out one as better than the rest, except on 4-domain proteins where DDomain and PDP excel.

**Table 4:** Comparison of the present work with previously published algorithms, ASTRAL30

**Algorithm**^**1**^	**1-domain**	**2-domain**	**3-domain**	**4-domain**	**Overall**^**2**^
SS Algorithm	75%	60%	47%	34%	70%
CA Algorithm	75%	58%	46%	33%	69%
DDomain	83%	58%	43%	44%	76%
DomainParser2	80%	56%	49%	25%	73%
PDP	74%	62%	49%	46%	70%
Random (see text)	76%	15%	2%	0%	60%

Present work compared to DDomain [12] (using AUTHORS parameters), DomainParser2 [13] and PDP [11]. ^1^CA refers to the α-carbon based algorithm and SS the secondary structure based one. ^2^Total, regardless of number of domains.

With optimization complete, the algorithms were then run on the Benchmark_2 test set. This set (see Methods) is significant in that the distribution of number of domains is intended to match that of the genome, and not the over-weighting of single domain proteins found in the PDB. Additionally, SCOP and CATH, as well as the structure authors, agree on the number of domains for all structures in this data set making the correct result less ambiguous. Note that this test set only contains 4 proteins with 4 domains, so reporting success rates for these is not statistically meaningful.

Table 5 compares the performance on Benchmark_2 to other published methods, and we find the CA algorithm is highly competitive (92% single-domain, 78% for 2-, 76% for 3- and 25% for 4-) at only 3% less overall than the best method (PDP) and roughly tied with DomainParser2. The random assigner performed significantly worse with averages of 71%, 15%, 1% and 0% correct for single, 2-, 3- and 4-domain proteins respectively (31% overall) over 3 trials. All the methods are clearly better than random. Again for DDomain, the AUTHORS settings were used. The SS algorithm does not fare as well on this set, performing significantly more poorly with success rates of 86%, 64%, 60% and 0% for 1-, 2-, 3-, and 4-domain proteins (69% overall). The overall rate of over-cutting for CA was 8.5% while for under-cutting it was 10.5%, comparable to that observed with the other methods except PDP which showed a stronger tendency to overcut rather than undercut (data not shown). The MCC for each assignment is also provided in Table 5, to again help compensate for the large bias towards single-domain structures in the data set. This produced the same ranking as the raw success rates however, if the 1-, 2- and 3-domain MCCs are just averaged. In terms of execution speed, the CA algorithm is over 15 times faster than either DomainParser2 or DDomain, and about 4 times faster than PDP, while the SS algorithm is faster than the CA by a further factor of 5.

**Table 5:** Comparison of the present work with previously published algorithms, Benchmark 2

**Algorithm**^**1**^	**Time**^**2**^	**1-domain**	**2-domain**	**3-domain**	**4-domain**	**Overall**^**3**^
SS Algorithm	5s	86% (0.75)	64% (0.47)	60% (0.46)	0% (−0.03)	69%
CA Algorithm	26s	92% (0.82)	78% (0.69)	76% (0.69)	25% (0.32)	80%
DDomain	497s	94% (0.78)	75% (0.68)	48% (0.56)	25% (0.16)	75%
DomainParser2	398s	98% (0.86)	75% (0.71)	64% (0.60)	50% (0.39)	79%
PDP	99s	92% (0.93)	84% (0.82)	68% (0.69)	75% (0.55)	83%
Random (see text)	1s	71 ± 3% (<0)	15 ± 7% (<0)	1 ± 2% (<0)	0% (<0)	31 ± 3%

Present work compared to DDomain [12] (using AUTHORS parameters), DomainParser2 [13] and PDP [11] on the Benchmark 2 data set. Matthews correlation coefficient is given in parentheses. ^1^CA refers to the α-carbon based algorithm and SS the secondary structure based one. ^2^Time taken for the actual execution of the binaries or detection algorithms over the full dataset on a single 2.93 GHz i7 CPU. ^3^Total, regardless of number of domains.

Lastly we tested the present methods on the Benchmark_3 set requiring 90% or better overlap. Benchmark_3 is a subset of the Benchmark_2 structures in which both SCOP and CATH also agree upon the exact boundaries of the domains, within a small tolerance, suggesting that the domain boundaries are sharply defined in this set. As seen in Table 6, the CA algorithm achieved 77% correct assignment (7% failure in overlap, 17% failure in domain number). Removing the constraint that prevents domain boundaries midway through secondary structure elements increased the performance to 79%, demonstrating that it is not always advisable to enforce this condition. Again the SS algorithm did not perform too well on this data set. The best method, PDP, did slightly better at 80% success. The MCC values show a similar trend in performance with CA just marginally behind DomainParser2 and PDP.

**Table 6:** Comparison of the present work with previously published algorithms, Benchmark 3

**Algorithm**^**1**^	**1-domain**	**2-domain**	**3-domain**	**4-domain**	**Overall**^**2**^
SS Algorithm	65% (0.70)	50% (0.39)	38% (0.31)	0% (−0.04)	53%
CA Algorithm	93% (0.87)	76% (0.71)	52% (0.59)	0% (−0.01)	77%
DDomain	94% (0.80)	66% (0.71)	43% (0.56)	33% (0.21)	74%
DomainParser2	96% (0.92)	71% (0.74)	67% (0.67)	67% (0.50)	79%
PDP	89% (0.93)	76% (0.82)	67% (0.71)	100% (0.77)	80%

Present work compared to DDomain [12] (using AUTHORS parameters), DomainParser2 [13] and PDP [11] on the Benchmark 3 data set. Matthews correlation coefficient is given in parentheses. ^1^CA refers to the α-carbon based algorithm and SS the secondary structure based one. ^2^Total, regardless of number of domains.

## Discussion

It is instructive to look at the types of mistakes made by the CA algorithm, which performed best overall on the test data sets, of the two methods developed in this work. There have already been detailed comparisons of SCOP and CATH published [21] so we will focus on the Benchmark_2 set where both databases agree. Of the 31 failures, only 2 were due to the overlap being less than 0.75 (and the number of domains otherwise correct). The 4 single domain proteins that were missed were assigned as 2- or 3-domain. There were also 7 2-domain proteins assigned as single domain, and another 5 assigned to have 3-domains. The other common error was assigning 3-domain proteins as 2-domain, with 4 occurrences. An example from each of these failure classes is shown in the following figures.

**Figure 1:**
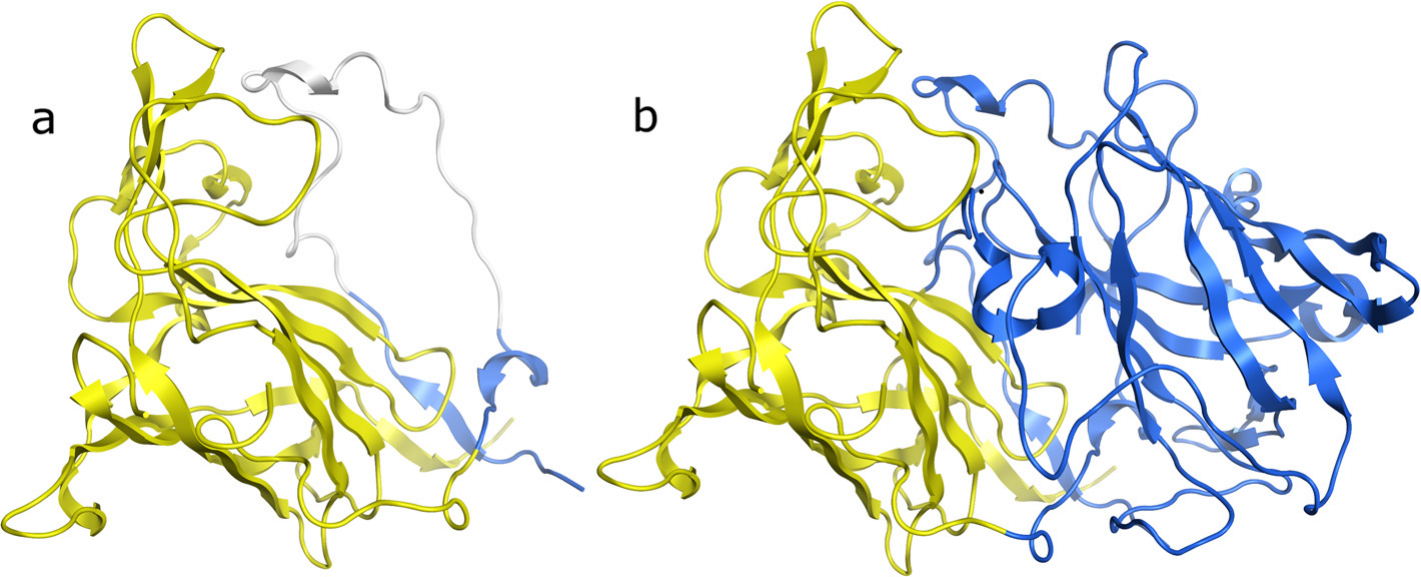
**PDB 2PCD domain assignments. a**) Chain M, assigned by the CA algorithm as two domains shown in yellow and blue. Gray region is too exposed and excluded from the assignment. **b**) Assignment run on the dimer of chains A and M together

2PCD chain M is a single domain assigned as two domains (Figure 1a). However the second domain (yellow) involves less than 10% of the chain and is in a very loopy region at the N-terminus which indeed is not close to anything else except a paired ß-strand at the C-terminus, also isolated from the rest of the protein. The present method does not pay any special attention to ß-strand pairing however, and perhaps enforcing that members of a single ß-sheet be in the same domain might improve the performance further. This particular structure is actually a dimer in nature (with chain A) [22], and so running our assigner on the dimer (Figure 1b) does indeed result in two domains: chain A and the first 50 residues of chain M form the first domain (blue), and the remainder of chain M the second (yellow). Thus the ‘second domain’ assigned for chain M was actually just part of the larger domain formed by chain A, its partner. This example highlights the potential danger of only looking at single chains for evaluating domain assignments. In this case ignoring chain A here causes a correct assignment to appear incorrect. Unfortunately most assignment methods cannot deal with domains spanning multiple chains, and so for the purposes of comparison and benchmarking, such a simplification is necessary. Ideally however, domain splitting should be performed on the full biological unit and we expect the present method to excel in its ability to do so. Over 54% of the Benchmark_2 structures are annotated as multimers by their authors however only 17 of the 31 failures (55%) occur in multimers so this does not appear to be the only factor with impact on the overall performance of the method. 

**Figure 2:**
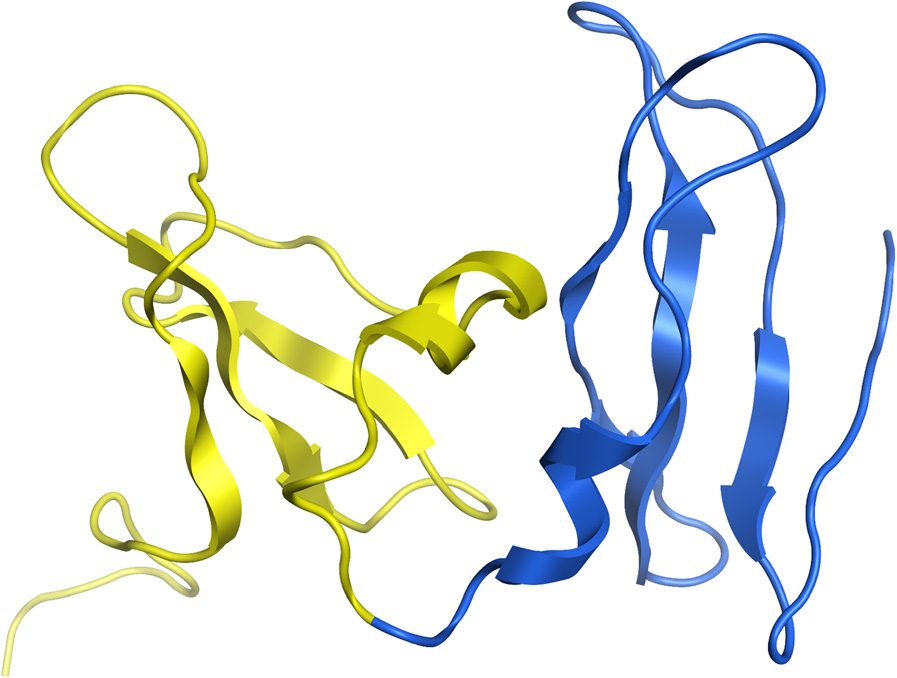
**PDB 1YUA chain A domain assignment.** It is assigned by the CA algorithm as single domain but is actually two domains, as shown in yellow and blue

A more clear failure of the algorithm is 1YUA chain A, which is a two domain protein assigned to be a single domain. Visually the protein is clearly two distinct domains, and the problem here is that they are just very small. Lowering our minimum cut value, m, to 19Å and running the assignment again (Figure 2) gets it exactly correct (but would get other examples incorrect). Our algorithm as parameterized is simply biased towards slightly larger domains than seen here, and so may produce incorrect assignments for very small domains.

**Figure 3:**
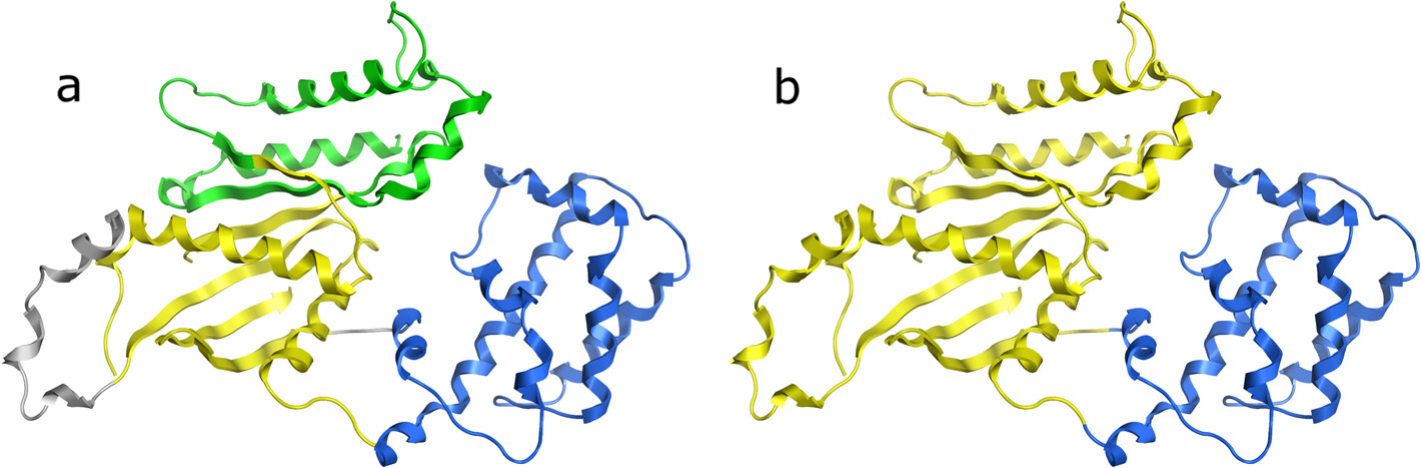
**PDB 1GDD chain A assignments. a**) Assigned by the CA algorithm as three domains. Gray region is exposed and excluded from the assignment. **b**) Actual domain assignment in Benchmark_2 set, two distinct domains

1GDD chain A is a two-domain protein assigned as three domains - the smaller domain location is assigned correctly, but the larger one is split in two (Figure 3). The SS algorithm correctly assigns two domains (and their cut points within 11 residues) so it is interesting to investigate why the CA algorithm decides on making an extra cut. Again this extra cut breaks up a six-stranded ß-sheet. It seems the lower density of Cαs around the sheet ‘fools’ the algorithm into splitting it up. Some sort of constraint to keep ß-sheets together would help - putting all Cαs within the same ß-sheet at distance 4Å from each other in the distance matrix results in a correct assignment of two domains (and perfect cut locations).

**Figure 4:**
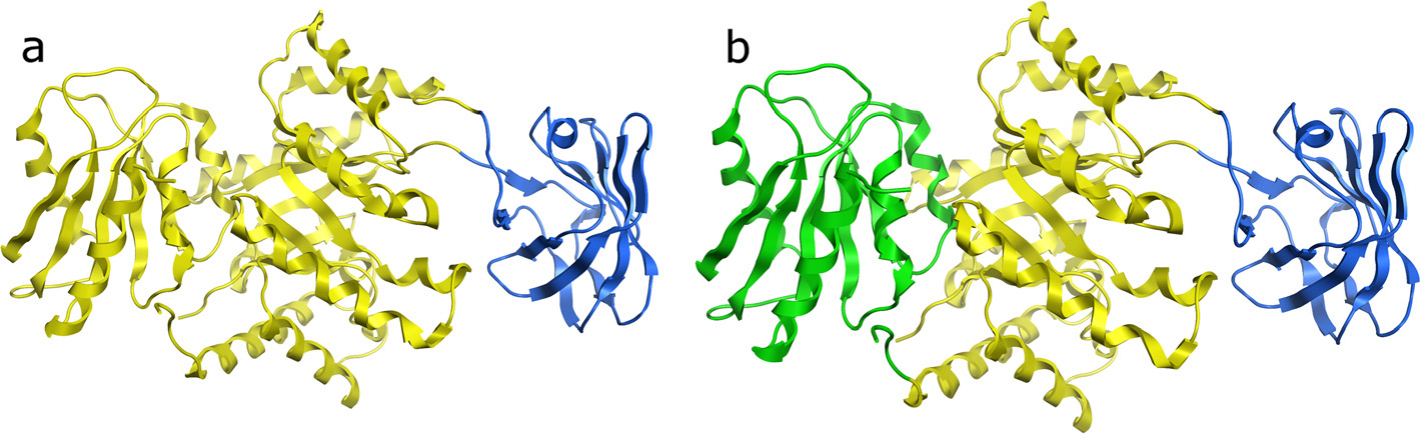
**PDB 1PKY domain assignments. a**) Chain A, assigned by the CA algorithm as two domains. **b**) Chain B, assigned by the CA algorithm as three domains, which matches exactly the correct split in the Benchmark_2 set

1PKY chain A is an example of a three-domain protein we assign as two-domain (Figure 4). This Pyruvate Kinase structure is a homo-tetramer. The CA algorithm here lumps the C-terminal domain together with the large central domain. However, using instead chain B results in a perfect split. Chains C and D are cut the same as chain A. The SS algorithm, which is less sensitive to small perturbations in coordinates since it only depends on the secondary structure elements, correctly splits all four chains into three domains. So in this case the CA algorithm proves to be too sensitive to the precise 3D coordinates used. Although the pairwise RMSD between chains A and B is only 0.43Å, this is apparently sufficient to make the difference between a correct and incorrect assignment - this is just an unfortunate borderline case and investigation of the clustering dendrogram (not shown) shows that this structure is close to the cutoff of m=22Å.

**Figure 5:**
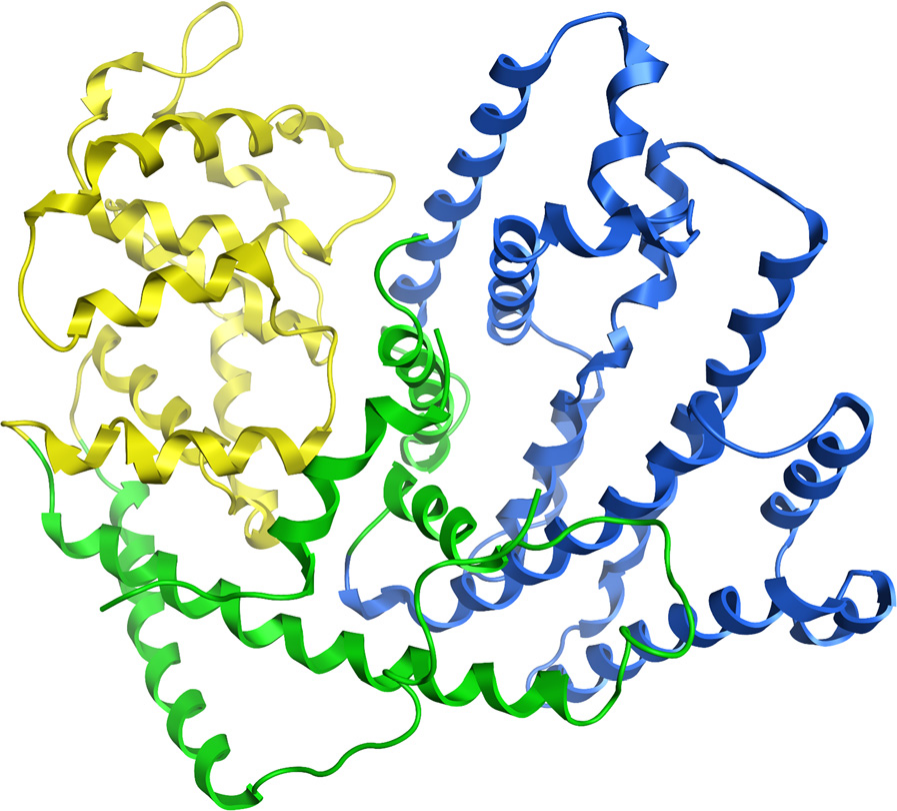
**PDB 5EAU chain A domains.** The portions of the two domains that the assignment by the CA algorithm and assignment in the Benchmark_2 set agree upon are shown in blue and yellow. The region shown in green is assigned to the yellow domain by the CA algorithm, but the blue domain by the data set

Finally, 5EAU chain A was correctly assigned as 2-domain but had an overlap of only 73% (Figure 5). This is a large all-helical protein, and while the cores of the two domains are essentially correct, it is the border region which is in dispute, shown in green in Figure 5. There is a long helix from residue 220–260 which serves to link the two domains together, and we assign it, along with a few neighboring helices, to one domain while SCOP and CATH assign it to the other. Interestingly the SS algorithm fares better on this one, with 89% overlap on its 2-domain assignments, only classifying the N-terminal helix in the ‘wrong’ domain (as per CATH) – this assignment for the helix does match SCOP however.

The above examples demonstrate several of the shortcomings of the CA algorithm, where improvement could be made in the future. It tends to perform best when the full biological assembly is provided, and may partition the complex differently depending how many copies of each chain are included. It is sensitive to quite small perturbations in coordinates for structures that are close to the cutting boundary (m); and for very small domains it will tend to undercut. DDomain, domainparser2 and PDP also fail mostly due to incorrect number of domains rather than overlap under 75%, and in each case roughly half the incorrect assignments overlap with the CA method’s failures. Thus the CA algorithm correctly assigns about half the failures of each of the other ones. In total 10 failed assignments are unique to the CA method including 2PCD, 1GDD and 5EAU above. Interestingly there are 5 structures that none of the algorithms assign correctly (1D0G chain T, 1DCE chain A, 1DGK chain N, 1KSI chain A and 2GLI chain A).

**Figure 6:**
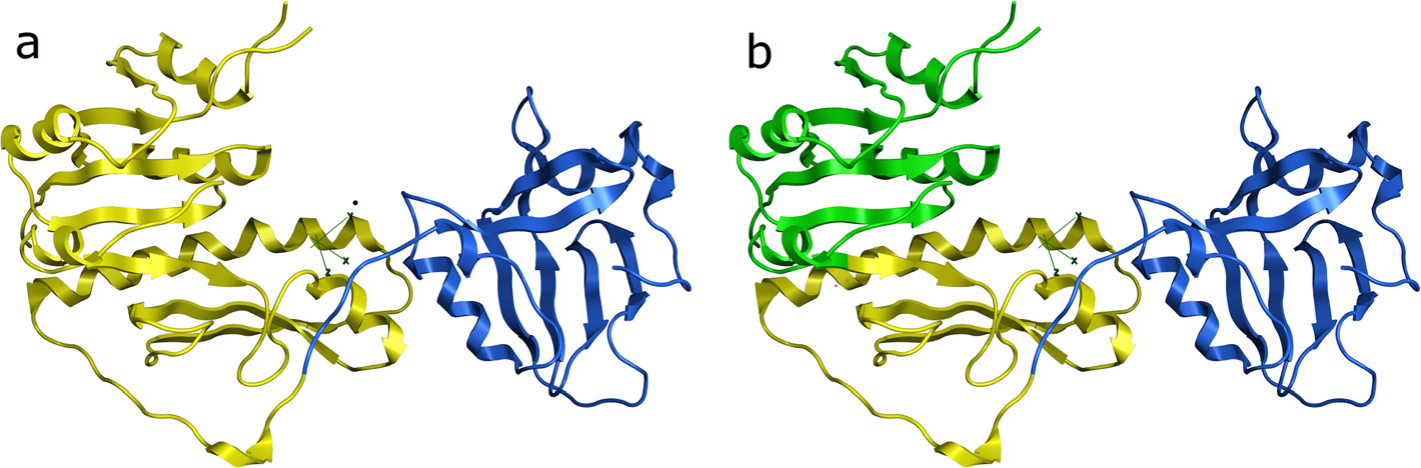
**PDB 1FMT chain A domain assignments. a**) Assignment by the CA algorithm as two domains, which matches exactly the correct split in the Benchmark_2 set. **b**) Incorrect assignment by DDomain, DomainParser2 and PDP. The extra incorrect domain is shown in green

An example where the CA algorithm assigned two domains correctly while the others all assigned three domains is 1FMT chain A (Figure 6). This is a monomeric tRNA formyltransferase protein, and although the split into 3 domains does not appear unreasonable visually, the two domains on the left of figure 6a are actually only one domain. It is not clear why the other programs all fail on this example, but it does demonstrate again that no one of the methods is always the most correct.

## Conclusions

This work presents two novel, related, domain assignment algorithms, one based on clustering buried α-carbons and one clustering secondary structure elements. They are appealing due to their intuitiveness, speed and extreme simplicity – having only two adjustable parameters – and are able to perform competitively with the best algorithms available. The CA algorithm is several times faster than other methods, and comes within a few percent of the top performer on all the data sets investigated, making its use appealing. It is worth noting that no one algorithm performed best on the ASTRAL30 *and* the Benchmark data sets. The algorithms in this study also have the advantage that they can be run on arbitrary numbers of chains, and have no artificial limitations on how many domains or segments they may assign. The CA algorithm is not limited to assigning cuts only at secondary structure boundaries, either.

The examples studied indicate that the CA algorithm should not be used when very small domains are expected. Also when multiple copies of a chain exist in the asymmetric unit, it should be run on each separately and perhaps the consensus assignment taken due to its sensitivity to small perturbations in coordinates. Keeping these limitations in mind, it is encouraging that such simple, fast methods can perform as well as they do.

Domain assignment within 3D protein structures is a difficult subject to tackle, due to it being an ill-defined problem to begin with. Different people have different definitions of what a domain is, and this definition might change depending on the intended application. Thus measuring the performance of a particular method, and comparing it to others, is difficult at best. That said, in some cases there is a clear and unambiguous split and the data sets from Holland *et al.*[23] go a long way towards providing a fair set to test on. The one important thing they have overlooked is the importance of considering the biological unit. Assignments need to be run on the full biological unit of a protein which should allow more accurate assignments for multimers, or else those structures which are not monomers should be further excluded from the test set.

Even the best methods are still far from perfect, and this is in part due to the subjective nature of the problem. With a problem like domain assignment, rather than focusing on which method is a few percent closer to SCOP or CATH, for example, it is perhaps more prudent to simply look at the cases where assignments differ from the ‘correct’ answer and ask ‘is this reasonable’?

## Methods

Two distinct though related algorithms were developed for this study, one using α-carbons and one secondary structure elements. Both methods attempt to cluster pieces of the structure using hierarchical agglomerative techniques.

### Alpha carbon algorithm

All α-carbons within the structure were identified, and all other atoms were ignored for the remainder of the process. The atoms were then divided into two sets: 'buried' and 'exposed'. Buried α-carbons were defined as those with 9 or more α-carbons within 7Å. These values were found empirically to correspond well to an intuitive definition of buriedness.

Next, an NxN pairwise distance matrix is constructed for all N buried α-carbons and these are clustered using average linkage clustering [24] to produce a dendrogram. Average linkage is a form of the more general hierarchical agglomerative clustering technique. Briefly, for a given set of N objects, and a distance matrix of their pairwise distances, objects are iteratively grouped two at a time to form larger and larger clusters. The pair with the shortest distance at each iteration is chosen for merging, and the distance of the newly formed cluster to existing clusters is computed based on the linkage employed. With average linkage, the distance between two clusters of objects is defined as the average distance between all pairwise combinations of objects within the two clusters. After N-1 iterations, a single cluster containing all N objects remains, along with a dendrogram with N-1 non-leaf nodes corresponding to the merges performed at each iteration.

**Figure 7:**
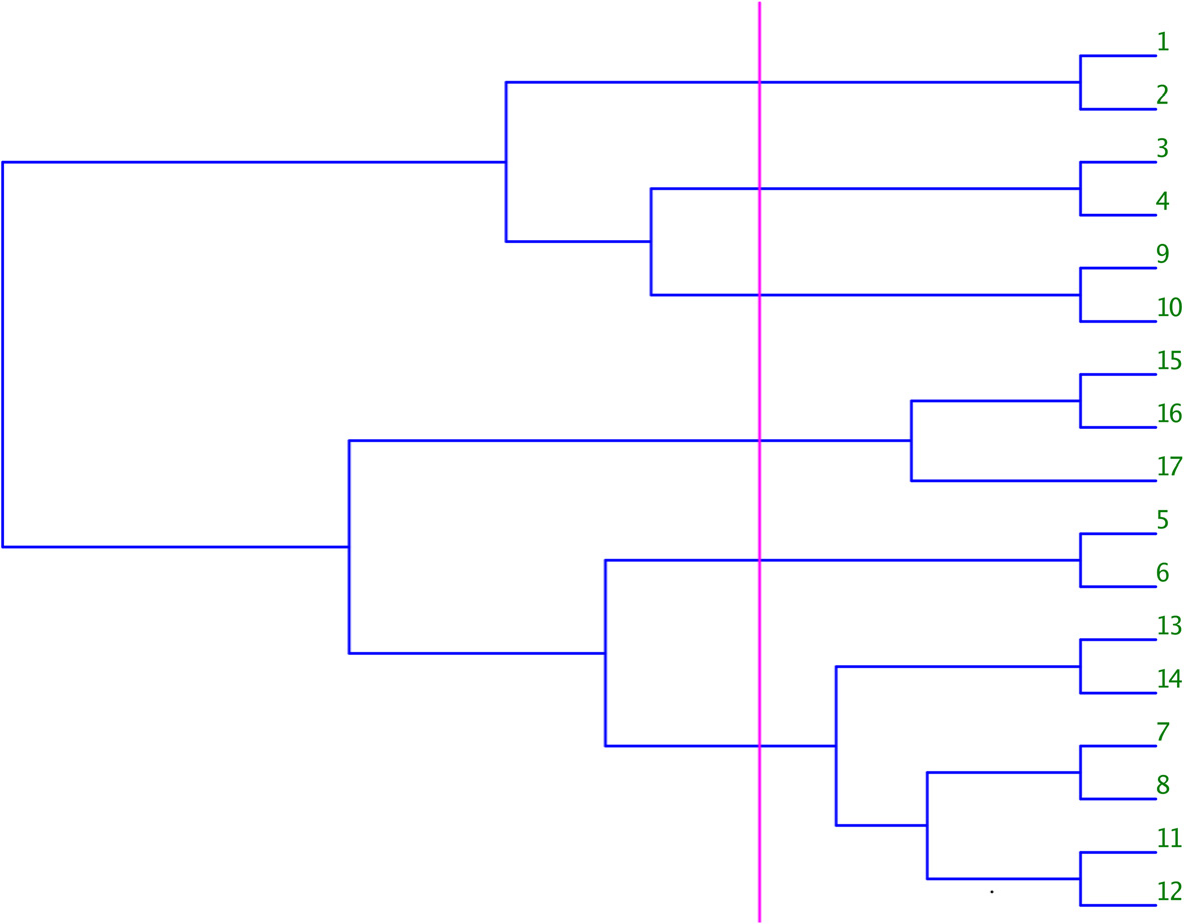
**Typical dendrogram resulting from average linkage clustering.** Original objects are numbered on the right, and a potential cut is shown in magenta. This particular cut would result in six clusters: (1, 2), (3, 4), (9, 10), (15, 16, 17), (5, 6) and (7, 8, 11, 12, 13, 14)

By cutting the dendrogram at a specific level, clusters of the original N objects are formed. Thus cutting this dendrogram at a specific point produces a number of clusters of α-carbons, which can then be defined as the domains. Obviously choosing where, and when, to cut the dendrogram is the key problem as this determines the number (and location) of domains (Figure 7). We define two parameters, m, the minimum domain size, and s, the step size. We refer to the distance axis along the dendrogram as d. To choose a cut point d=D, we proceed as follows:

1. Start at D = max d, the root of the dendrogram

2. If D < m then stop without making a cut

3. If no branch node of the dendrogram is traversed between D and D - s, stop and make the cut

4. Set D = d’, the value at the branch node traversed in the previous step

5. Return to step 2 and repeat

Thus the algorithm seeks to cut at a region of clear separation in the dendrogram, but not making the domains too small. These parameters were optimized on a number of test sets as described in Results, and values of m = 22Å and s = 5Å produced the best results.

Residues which were initially classified as exposed are at this point added to the cluster of the nearest buried atom. Clustering was also tested on all α-carbons, but using just the buried ones both tended to produce better results, and was also faster, there being less points to cluster.

Lastly, a bit of 'clean-up' is performed. This clustering technique can sometimes result in some small clusters of just a few residues being created, and these were eliminated by simply deleting any clusters less than 10% of the size of the largest cluster. Also, because no heed is paid to chain or residue sequence, the algorithm would frequently produce small stretches of a few amino acids from one domain, within the sequence of another when adding back the exposed α-carbons to the clusters. In order to try to minimize the number of small segments like this, the sequence is scanned linearly for segments less than 20 residues in length. Any such short segments which are enclosed on both sides by residues of the same cluster, or which appear at the ends of a chain, are consumed by the adjacent cluster and become a part of it.

A minor variation on the algorithm was tested which helped prevent placing domain boundaries midway through secondary structure elements. When building the pairwise distance matrix, all residues pairs which were within the same secondary structure element, as defined by DSSP [25], were given a distance of 4Å - roughly the distance between adjacent α-carbons along the backbone. While this did provide a slight improvement in performance on the test set, this may be considered a limitation rather than an advantage and so is left up to the discretion of the user whether to make use of it or not. It was used in all results presented here unless noted otherwise.

### Secondary structure algorithm

We also experimented with a routine that looked only at secondary structure elements. Its performance was comparable to the α-carbon approach and was faster, there being less objects to cluster. Ultimately as shown in Results, the α-carbon method was found to be superior, and preferable, being independent of any particular secondary structure definition.

**Figure 8:**
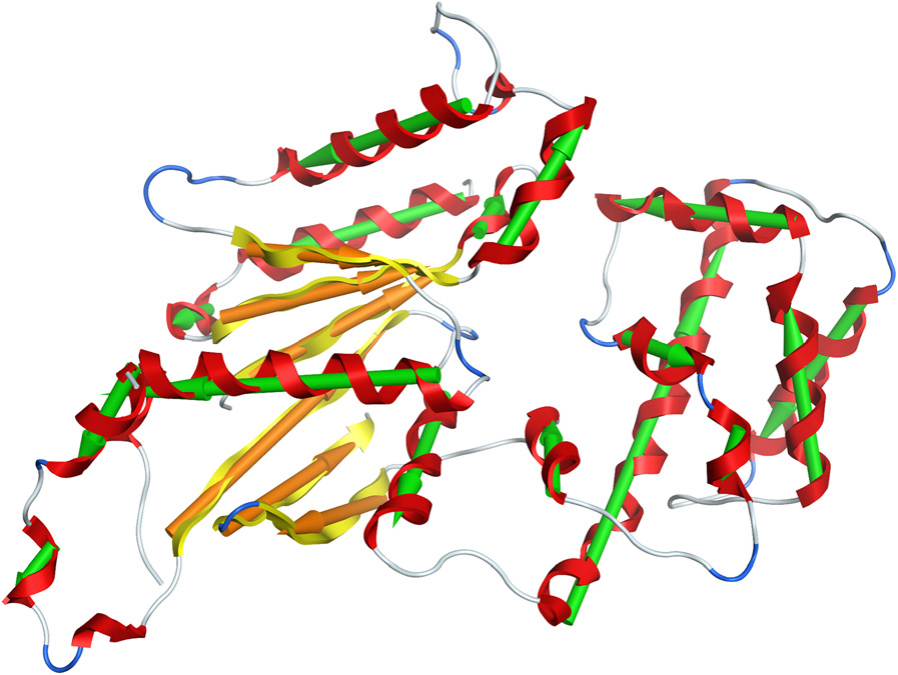
**PDB 1GDD with vector representation used by the SS algorithm shown.** Vectors representing helices are in green, and those representing strands are orange. The direction of the vector in each case is from N- to C-terminus

First secondary structure elements are identified, using DSSP. Elements are represented by vectors, with direction computed as the largest eigenvector of the covariance matrix of the Cα coordinates comprising the helix or strand (Figure 8). We denote this direction by a unit vector, $\vec{v}$. The center of mass of the element is found by simply averaging the atom coordinates, and is denoted by $\vec{c}$. Thus if ${\vec{r}}_{1}$ is the position of the first α-carbon in the helix or sheet, then the start of the secondary structure vector is given by $\vec{c}\;+\;\left(\left({\vec{r}}_{1}\;-\;\vec{c}\right),\cdot ,\;,\vec{v}\right)\vec{v}$, and similarly the end is given by $\vec{c}\;+\;\left(\left({\vec{r}}_{2}\;-\;\vec{c}\right),\cdot ,\;,\vec{v}\right)\vec{v}$, where ${\vec{r}}_{2}$ is the position of the last Cα in the secondary structure element.

A special check is made for elements spanning chain breaks - these are broken into two elements, one on either side of the break. Although helices and β-strands often curve, the curve is usually gentle and we found that they are represented sufficiently well by a single vector.

Next, as in the previous algorithm, an NxN distance matrix is constructed. Here the distance between two secondary structure elements was defined either as the distance of closest approach of the two corresponding secondary structure vectors, or as the distance between the centers of the vectors. In practice the latter was found to work better. Again average linkage clustering was employed to produce a dendrogram, and the same procedure as in the previous algorithm was used to determine where and if to cut the dendrogram to produce clusters of the secondary structure elements. In this case m = 22Å and s = 5Å were found to be the optimal values, interestingly the same values used for the α-carbon algorithm despite the fact that much larger objects were now being clustered.

As before, very small domains are undesirable so all clusters of one or two secondary structure elements were discarded. Lastly, domain cut points were defined midway along the sequence between consecutive secondary structure elements that belonged to different clusters. This choice is somewhat arbitrary but usually produces satisfactory results.

A variation to this algorithm which obtained slightly improved results, was to mark secondary structure elements that were adjacent in sequence space as having a distance of 4Å when constructing the distance matrix before clustering. This was analogous to the variation in the α-carbon algorithm where those atoms in the same helix or sheet were set to have a distance of 4Å in that distance matrix. This modification tended to keep consecutive elements within the same cluster unless there was a good reason not to, and thus resulted overall in fewer disjoint segments among the assignments.

Both algorithms have been implemented within MOE [26] using the SVL programming language. Source code is available as supplemental information. Average run times for a single protein chain were 45ms for the CA algorithm and 9ms for SS, on a single 3 GHz CPU. The majority of the time was spent building the cluster dendrogram.

### Data sets

As mentioned earlier, there is no ideal test set for domain assignment, which makes it difficult to evaluate the performance in an unbiased manner. Holland *et al.*[23] have published an extensive comparison of several domain splitting algorithms and derived several Benchmark data sets used for the evaluation. Specifically, the Benchmark_2 data set was chosen with several points in mind: a) the PDB has a heavy bias towards single domain proteins - this data set was chosen to avoid this and to reflect the true distribution in the genome; b) only chains where SCOP, CATH and the authors of the X-ray or NMR structure agree on the number of domains were included; and c) at least one domain in each chain had to represent a unique CATH Topology class in the data set for that chain to be included, ensuring a diverse set of structures. This data set does not include genetic domains - that is, all domains are contained within a single protein chain. Though not entirely clear, it appears domain boundary locations were taken from CATH in this data set. A stricter set was also created by the same authors, Benchmark_3, which further removed those chains where domain boundaries differed between SCOP and CATH. The Benchmark sets thus represent an unbiased set of domains which are fairly unambiguous in definition, allowing them to be used to compare different domain assignment methods without worrying about the subjectivity sometimes involved in domain assignment. Only half the Benchmark_2 and Benchmark_3 data sets are made available for download for a total of 156 and 135 chains, respectively.

Additionally, a second much larger data set, ASTRAL30, was used. This is a non-redundant set of SCOP domains with no more than 30% sequence identity between any two domains. The entire chain, for all chains with at least one domain in the ASTRAL30 set, was included for the purposes of this work. For this data set, when SCOP and CATH disagreed on domain assignment, SCOP was chosen as the ‘correct’ one except where otherwise noted. This set is heavily biased, with 75% of the 7076 chains having a single domain.

In this work, if an assignment had a different number of domains than the value in the test set, it was considered incorrect. When the number of domains matched, a procedure similar to that described in Holland *et al.*[23] was used to determine correctness. Briefly, all possible permutations mapping domains from the assignment to those in the test set assignment were computed, and in each case, the overlap computed. Overlap is simply the number of residues assigned to the same domain number in the assignment and in the test set, divided by the total number of residues. The permutation producing the highest overlap is chosen as the correct mapping. Unless otherwise stated, an overlap of 75% or higher was required for an assignment to be considered correct.

## Competing interests

The author declares that they have no competing interests.

## Author contribution

HF performed all tasks.
